# The PI3K/AKT/mTOR signaling pathway is aberrantly activated in primary central nervous system lymphoma and correlated with a poor prognosis

**DOI:** 10.1186/s12885-022-09275-z

**Published:** 2022-02-20

**Authors:** Xiaowei Zhang, Yuchen Wu, Xuefei Sun, Qu Cui, Xueyan Bai, Gehong Dong, Zifen Gao, Yaming Wang, Chunji Gao, Shengjun Sun, Nan Ji, Yuanbo Liu

**Affiliations:** 1 Department of Hematology, Beijing Tiantan Hospital, Capital Medical University, Nan Si Huan Xi Lu 119, Fengtai District, Beijing, 100070 China; 2 Department of Hematology, Beijing Shijitan Hospital, Capital Medical University, Beijing, China; 3 Department of Pathology, Beijing Tiantan Hospital, Capital Medical University, Beijing, China; 4 Department of Neurosurgery, Xuanwu Hospital, Capital Medical University, Beijing, China; 5 Department of Hematology, Chinese PLA General Hospital, Beijing, China; 6 Neuroimaging Center, Beijing Tiantan Hospital, Capital Medical University, Beijing, China; 7 Department of Neurosurgery, Beijing Tiantan Hospital, Capital Medical University, Beijing, China

**Keywords:** Primary central nervous system lymphoma, PI3K/AKT/mTOR signaling pathway, *PTEN*, Prognosis

## Abstract

**Background:**

Primary central nervous system lymphoma (PCNSL) is a specific subtype of non-Hodgkin lymphoma that is highly invasive and confined to the central nervous system (CNS). The vast majority of PCNSLs are diffuse large B-cell lymphomas (DLBCLs). PCNSL is a highly heterogeneous disease, and its pathogenesis has not yet been fully elucidated. Further studies are needed to guide individualized therapy and improve the prognosis.

**Methods:**

In this study, we detected 1) the expression of p-AKT, p-mTOR, p-S6 and p-4E-BP1 by immunohistochemistry (IHC) and Western blotting, 2) the mRNA expression by real-time qPCR and 3) the deletion of *PTEN* gene by immunofluorescence in situ hybridization (FISH) in order to investigate the activation status of the PI3K/AKT/mTOR signaling pathway in PCNSL. Samples of reactive hyperplasia lymphnods were used as the control group. The correlations between the clinical characteristics and prognosis of PCNSL patients and the expression of p-AKT, p-mTOR, p-S6 and p-4E-BP1 and the deletion of *PTEN* were assessed.

**Results:**

The IHC results showed that the positive expression rates of p-AKT, p-mTOR, p-S6 and p-4E-BP1 in PCNSL were significantly higher in the PCNSL group than in the control group (*P* < 0.05). The relative mRNA expression level of *MTOR* in PCNSL samples was significantly increased (*P* = 0.013). Correlation analysis revealed that the expression of p-mTOR was correlated with that of p-AKT, p-S6, p-4E-BP1. *PTEN* deletion was found in 18.9% of PCNSL samples and was correlated with the expression of p-AKT (*P* = 0.031). Correlation analysis revealed that the PCNSL relapse rate in the p-mTOR-positive group was 64.5%, significantly higher than that in the negative group (*P* = 0.001). Kaplan-Meier survival analysis showed inferior progression-free survival (PFS) in the p-mTOR- and p-S6-positive groups (*P* = 0.002 and 0.009, respectively), and *PTEN* deletion tended to be related to shorter overall survival (OS) (*P* = 0.072). Cox regression analysis revealed p-mTOR expression as an independent prognostic factor for a shorter PFS (hazard ratio (HR) =7.849, *P* = 0.046).

**Conclusions:**

Our results suggest that the PI3K/AKT/mTOR signaling pathway is aberrantly activated in PCNSL and associated with a poor prognosis, which might indicate new therapeutic targets and prognostic factors.

**Supplementary Information:**

The online version contains supplementary material available at 10.1186/s12885-022-09275-z.

## Background

Primary central nervous system lymphoma (PCNSL) is a rare and aggressive type of extranodal non-Hodgkin lymphoma that is confined to the central nervous system (CNS), which includes the brain, leptomeninges, eyes and spinal cord, and has no involvement of peripheral tissues and organs at the time of diagnosis. The main pathological type of PCNSL is diffuse large B-cell lymphoma (DLBCL), accounting for approximately 95% of PCNSL cases [1]. With the clinical application of high-dose methotrexate-based immunochemotherapy, the remission rate of PCNSL patients has significantly improved [2]. However, compared to systemic DLBCL, the efficacy and prognosis of PCNSL are still unsatisfactory [3, 4]. The high risk of relapse and early mortality remains a serious threat to patients’ long-term survival [5, 6].

Over the past four decades, the incidence of PCNSL has apparently increased in immunocompetent populations, especially in patients over 70 years of age [7]. Epstein-Barr virus (EBV) infection is a crucial oncogenic factor in immunocompromised patients with PCNSL [8, 9]. Both acquired and congenital immunosuppression have been shown to be susceptibility factors for PCNSL [10]. However, in immunocompetent patients, the pathogenesis of PCNSL is more complicated. Recent studies revealed that activation of the B cell receptor (BCR) and NF-κB pathway caused by mutations of myeloid differentiation factor 88 (MyD88) and CD79B is an important mechanism of the occurrence and development of PCNSL [11–15]. However, as a highly heterogeneous disease, the pathogenesis of PCNSL is quite complex. Recent studies have shown that various factors can drive the occurrence of PCNSL [16–19]. Therefore, further studies on the mechanisms of tumorigenesis of PCNSL are needed to better guide individualized treatment and prolong the survival of patients.

The PI3K/AKT/mTOR signaling pathway is one of the most important pathways in humans and widely exists in different tissues. It controls the basic physiological activities of tissue cells, such as the growth, proliferation, differentiation, apoptosis and metabolism of cells. In addition, it also plays an important role in the occurrence and development of malignant tumors and has become a potential therapeutic target. The research and development of PI3K/AKT/mTOR signaling pathway-related inhibitors and the clinical application evaluation in tumor therapy are current research hot spots.

Recently, several studies have found that the PI3K/AKT/mTOR signaling pathway is aberrantly activated in systemic DLBCL, participating in the occurrence and development of DLBCL [20–24]. The antitumor effect of the relevant inhibitor was also verified in cell and animal models [25–27]. In addition, the PI3K/AKT/mTOR signaling pathway could be activated by BCR activation [28, 29], indicating there might be crosstalk between these two pathways. Unfortunately, there are few studies studying this pathway in PCNSL at present. Therefore, it is worth further investigating the roles of the PI3K/AKT/mTOR signaling pathway in PCNSL.

## Methods

In this study, we discussed the activation status of the PI3K/AKT/mTOR signaling pathway in PCNSL at the protein and transcript levels and preliminary discussed the role of the *PTEN* gene in the activation mechanism of this signaling pathway. In addition, we also analyzed the relationship between the activation of the PI3K/AKT/mTOR signaling pathway and the clinical characteristics and prognosis of patients with PCNSL in order to provide new ideas for exploring novel treatment strategies and potential indicators for prognosis evaluation.

### Patients and tissue samples

Tumor samples were acquired by surgical resection or stereotactic biopsy of PCNSL in 43 immunocompetent patients who were newly diagnosed and treated at Beijing Tiantan Hospital, Capital Medical University from December 2015 to February 2020. All 43 patient specimens were fixed with formalin. Since most tumor specimens were obtained by stereotactic biopsy, only 7 patients were able to have excess tissue specimens stored at − 80 °C. All the patients met the following criteria: 1) age over 18; 2) no glucocorticoid treatment before the tumor tissues were obtained; 3) at least 3 cycles of high-dose methotrexate-based induction chemotherapy after diagnosis; 4) no acquired and congenital immunosuppression situation. The patients were followed up until December 2020. Clinical characteristics, including age, sex, Eastern Cooperative Oncology Group (ECOG) score, lactate dehydrogenase (LDH) at initial diagnosis, number of lesions, deep brain involvement, pathological subtypes, recurrence, and survival outcome, were collected for all 43 patients. The progression-free survival (PFS) and overall survival (OS) of the patients were assessed. In addition, 6 samples of reactive hyperplastic lymph nodes were collected randomly as the control group.

### Immunohistochemistry (IHC)

The tissue samples were fixed with formalin and embedded in paraffin to make blocks. These paraffin-embedded samples were cut into 4-μm sections. After dewaxing with xylene and rehydration with graded ethanol, the sections were heated for antigen retrieval in citrate buffer (10 mM, pH 6.0) in a microwave. Then, 0.3% hydrogen peroxide was used to inactivate endogenous peroxidases, and 10% goat serum was used to block the nonspecific binding sites. After washing with PBS, sections were incubated overnight at 4 °C with the following primary antibodies: p-AKT^Ser473^ (1:100 dilution, Cell Signaling Technology (CST), Beverly, MA, USA), p-mTOR^Ser2448^ (1:50 dilution, Abcam, Cambridge, UK), p-S6^Ser240/244^ (1:2000 dilution, CST) and p-4E-BP1^Thr37/46^ (1:1600 dilution, CST). The next day, these sections were incubated with the working solution of HRP-linked secondary antibody at room temperature for 1 h and then developed with 3,3′-diaminobenzidine (DAB) solution. Finally, the sections were counterstained with hematoxylin. The slides were digitally scanned using Vectra Polaris slide scanner (PerkinElmer) at 40x magnification. The whole slide images were processed using Phenochart software. Two independent pathologists, who were blind to the clinical data, evaluated the staining results from five randomly selected high-power fields.

Positive staining was assessed according to the intensity and the range of positive staining. The intensity was scored from 0 to 2 (0 for absent staining, 1 for weak staining, and 2 for moderate or strong staining). The range was indicated by the percentage of positive staining, scored from 1 to 3 (1 for < 10%, 2 for 10–50%, 3 for > 50%) [30]. Generally, protein expression was considered positive if the product of the intensity and range scores was greater than 3.

### Western blotting

Western blotting was performed on frozen tissue samples preserved at − 80 °C. We used RIPA lysis buffer, proteinase inhibitors and phosphatase inhibitors as the protein extraction reagents. A bicinchoninic acid (BCA) protein analysis kit was employed to examine the protein concentration of each sample. The proteins of the samples were loaded onto sodium dodecyl sulfate (SDS) polyacrylamide gels and transferred to polyvinylidene fluoride (PVDF) membranes after electrophoresis. In general, the membranes were subsequently blocked by using 5% skimmed milk and incubated for 1 h at room temperature under agitation. For phosphorylated protein detection, we used 5% bovine serum albumin (BSA) for blocking. Then, the membranes were incubated overnight at 4 °C with the following primary antibodies: pan-AKT (CST), p-AKT^Ser473^ (CST), m-TOR (CST), p-mTOR^Ser2448^ (Abcam), S6 (CST), p-S6^Ser240/244^ (CST), 4E-BP1 (CST) and p-4E-BP1^Thr37/46^ (CST) at a dilution of 1:1000. β-Actin was used as an internal reference. After that, the membrane was incubated with HRP-linked secondary antibodies at room temperature for 1 h. Finally, the membrane was exposed to film using GBox-Chemi XX9 (Syngene) after being incubated with enhanced chemiluminescence substrate. We used GeneTools software to evaluate the grayscale intensity and perform quantitative analysis of the extracted protein.

### Quantitative real-time PCR (qPCR)

Total RNA was isolated using TRIzol and converted to cDNA using the Transcriptor First Strand cDNA Synthesis Kit (Roche) according to the manufacturer’s protocol. The DNA primers for the *AKT1, MTOR, RPS6* and *EIF4EBP1* genes were designed with Primer-BLAST software, and the sequences of each primer are listed in Supplementary Table S1. We chose PowerUp SYBR Green Master Mix (Applied Biosystems) as the reagent. qPCR assays were run on 7500 Fast Real-Time PCR System (Applied Biosystem). The relative expression levels of each target gene were determined by the 2^-△△CT^ method.

### Fluorescence in situ hybridization (FISH)

In this study, we conducted FISH to detect the deletion of the *PTEN* gene in PCNSL samples. Formalin-fixed, paraffin-embedded (FFPE) tissue blocks were sectioned at 4-μm thickness, deparaffinized, and rehydrated in xylene and graded ethanol. Subsequently, the slides were placed in the pretreatment reagent (Abbott Vysis) for tissue digestion for approximately 45 min. The digestion time was adjusted appropriately according to the degree of tissue digestion. The slides were then mixed with PTEN (10q23) probe (Abbott Vysis) according to the instructions of the manufacturer, placed into a hybridization system (Abbott Molecular Thermobrite Stat Spin), denatured for 20 min at 75 °C and incubated overnight at 37 °C. After washing the slides with saline-sodium citrate (SSC) buffer and IGEPAL (Sigma) to remove nonspecific background signals, the slides were counterstained with diamidino-2-phenylindole (DAPI) for fluorescence microscopy observation. The slides were scanned using LSM-880 laser-scanning confocal microscope (Zeiss). The images were processed with ZEN software. Five randomly selected high-magnification fields were used to analyze at least 100 interphase nuclei for each PCNSL sample. The *PTEN* gene was considered deleted in the sample if the ratio of the gene probe to the centromere probe was less than 0.8 [31].

### Statistical analysis

Statistical analysis was performed with SPSS 26.0 software. Two-tailed t-tests were used for parameter analysis. The frequency analysis of the nominal data was performed by Pearson’s χ^2^ test or two-tailed Fisher’s exact test. The Pearson correlation coefficient or Spearman rank correlation coefficient was used for correlation analysis. Kaplan-Meier curves were used for survival analysis, and the log-rank test was used to determine the significance for survival comparisons. The prognostic factors were analyzed by a multivariate Cox proportional hazards regression model. Differences with *P* < 0.05 were considered statistically significant.

## Results

### Expression of p-AKT, p-mTOR, p-S6 and p-4E-BP1 in PCNSL

**Fig. 1:**
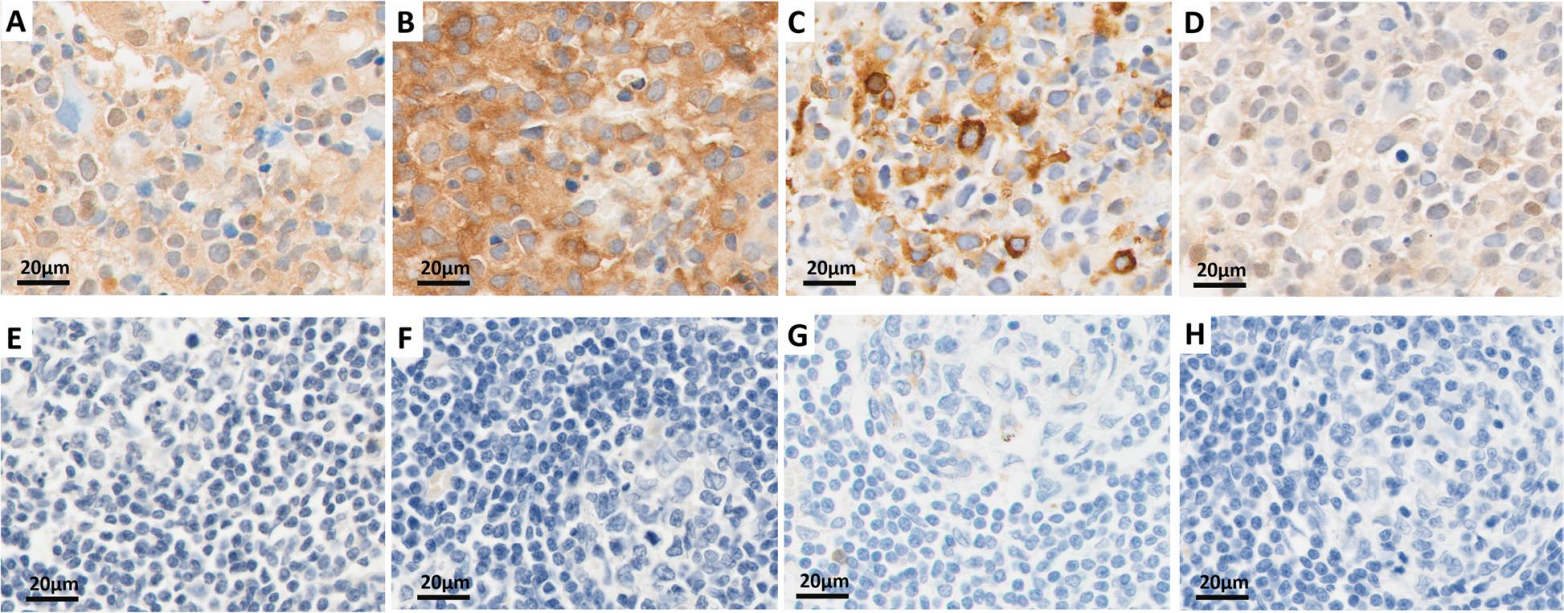
Immunohistochemical staining showed that p-AKT, p-mTOR, p-S6 and p-4E-BP1 were widely expressed in PCNSL samples (**A-D**, *n* = 43) but not expressed in reactive hyperplastic lymph node samples (**E-H**, *n* = 6) (hematoxylin counterstain, × 40)

Immunohistochemical staining was performed on 43 PCNSL tissue specimens and 6 reactive hyperplastic lymph node specimens (the control group). The staining results showed positive staining in brown or brownish-yellow colors. For the p-AKT and p-mTOR proteins, positive staining was mainly located in the cytoplasm. The p-S6 protein was mainly expressed around the nucleus. The p-4E-BP1 protein was mainly expressed in both the nucleus and cytoplasm. The IHC results of the above proteins are shown in Fig. 1.

p-AKT was widely expressed in the cytoplasm of 43 PCNSL tissue samples, with a positive expression rate of 65.1% (28/43). In control group, p-AKT was detected in only one sample, with a positive rate of 16.7% (1/6). The difference was statistically significant, with a *P* value of 0.035.

For p-mTOR, the positive expression rate was 72.1% (31/43) in the PCNSL tissue samples versus 16.7% (1/6) in the control samples, with a P value of 0.027. The only lymph node sample with p-mTOR positivity was the same sample that expressed p-AKT, as mentioned above.

The positive rates of p-S6 and p-4E-BP1 in PCNSL tissues were 79.1% (34/43) and 72.1% (31/43), respectively. However, there were only 2 samples that were p-S6-positive in the control group, and no obvious positive staining of p-4E-BP1 was observed. The positive rates of p-S6 and p-4E-BP1 in the PCNSL group were significantly higher than those in the control group, and the differences were statistically significant (*P* = 0.036 and 0.001, respectively).

**Fig. 2:**
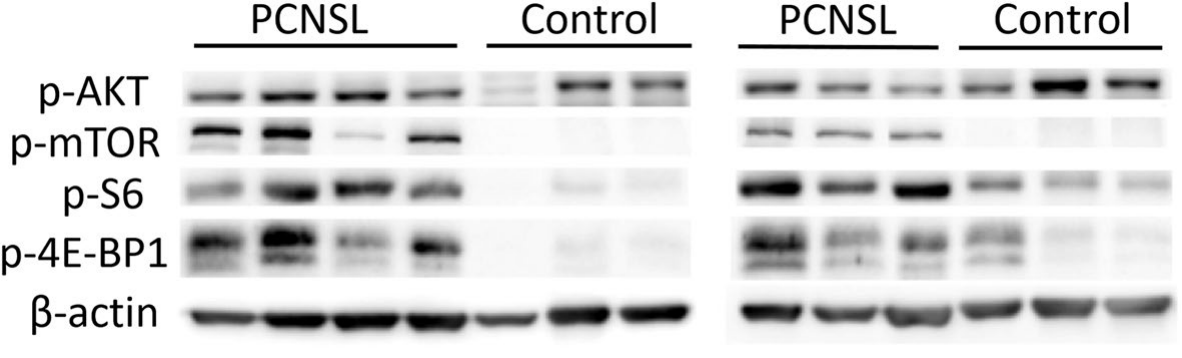
The expression levels of p-AKT, p-mTOR, p-S6 and p-4E-BP1 in PCNSL samples (*n* = 7) and reactive hyperplastic lymph node samples (the control group, *n* = 6) were detected by Western blotting. The quantitative analysis of the proteins was performed using GeneTools software. Full-length blots are presented in Supplementary Figure 1

In addition to IHC, we used Western blotting to further confirm the expression of the abovementioned phosphorylated proteins in PCNSL. The results are shown in Fig. 2. The grayscale values of each sample band after internal reference correction were measured and calculated. The differences in the expression levels of each targeted protein between the PCNSL group (*n* = 7) and the control group (*n* = 6) of samples were analyzed by t-tests. The results indicated that the expression levels of p-mTOR, p-S6 and p-4E-BP1 in the PCNSL samples were significantly higher than those in the reactive hyperplastic lymph node samples, and the differences were statistically significant (Table 1). The results were roughly consistent with the results of IHC.

**Table 1:** Comparison of p-AKT, p-mTOR, p-S6, and p-4E-BP1 expression levels between PCNSL samples and reactive hyperplastic lymph node samples (the control group)

Protein	PCNSL	Control	*P* value
	(*n* = 7)	(*n* = 6)	
p-AKT	0.352 ± 0.104	0.346 ± 0.168	0.935
p-mTOR	0.489 ± 0.268	0.065 ± 0.030	0.006*
p-S6	0.986 ± 0.324	0.378 ± 0.344	0.007*
p-4E-BP1	1.557 ± 0.668	0.526 ± 0.592	0.014*

Data are shown as the mean ± SD; * represents a statistically significant difference

In conclusion, the IHC and Western blotting results demonstrated that p-AKT, p-mTOR, p-S6 and p-4E-BP1 were overexpressed in the PCNSL group compared to the reactive hyperplastic lymph node group. Since these proteins are all key molecules in the PI3K/AKT/mTOR signaling pathway, we speculated that this pathway is abnormally activated in PCNSL.

### Correlation of p-mTOR expression with p-AKT, p-S6 and p-4E-BP1 expression in PCNSL

According to the results of immunohistochemical staining of 43 PCNSLs, Spearman rank correlation coefficient analysis showed that p-mTOR expression was correlated with p-AKT, p-S6, and p-4E-BP1 expression, with correlation coefficients of 0.415, 0.445, and 0.422, respectively. The calculated *P* values were 0.006, 0.003, and 0.005, respectively (all < 0.05), showing statistical significance.

The gray values of total protein and phosphorylated protein of mTOR, AKT, S6 and 4E-BP1 in 7 PCNSL samples were measured simultaneously by Western blotting. The ratio of the grayscale value of the phosphorylated protein to that of the total protein was calculated to evaluate the phosphorylation level of each protein. Pearson correlation coefficients were used to analyze the relationship of the phosphorylation level of mTOR with that of AKT, S6, and 4E-BP1. The results showed that the correlation coefficient between the phosphorylation level of mTOR and S6 was 0.871 with a *P* value of 0.011, which was statistically significant. However, there was no significant correlation between mTOR phosphorylation level and the other two proteins (*P* > 0.05).

### mRNA expression of AKT1, MTOR, RPS6 and eIF4EBP1 genes in PCNSL

**Fig. 3:**
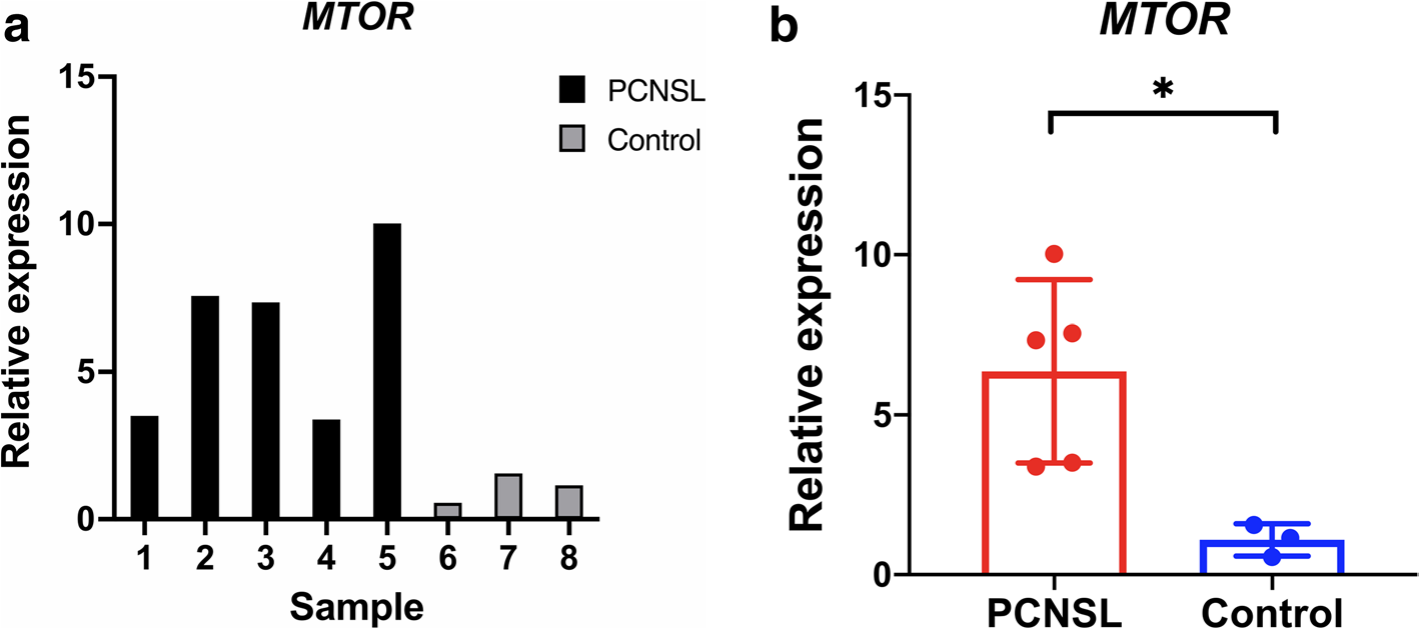
Relative mRNA expression of *MTOR* in the PCNSL (*n* = 5) and control (*n* = 3) groups. The relative mRNA expression level of *MTOR* in PCNSL samples was significantly higher than that in the control group

We selected the following related genes, *AKT1, MTOR, RPS6* and *EIF4EBP1*, and detected their mRNA expression in 5 samples of PCNSL by real-time qPCR. Three samples of reactive hyperplastic lymph nodes were used as the control group. The relative mRNA expression levels of these target genes are shown in Table 2. The results showed that the relative mRNA expression level of *MTOR* in PCNSL samples was 6.363 ± 2.864, which was significantly higher than the 1.089 ± 0.503 value in the control group; the *P* value was 0.013 (Fig. 3).

**Table 2:** Comparison of the relative mRNA expression levels of AKT1, MTOR, RPS6 and eIF4EBP1 in the PCNSL and control groups

Gene	PCNSL	Control	*P* value
	(*n* = 5)	(*n* = 3)	
*AKT1*	1.252 ± 0.766	1.245 ± 0.624	0.817
*MTOR*	6.363 ± 2.864	1.089 ± 0.503	0.013*
*RPS6*	3.359 ± 4.570	1.031 ± 0.307	0.426
*EIF4EBP1*	0.998 ± 0.349	1.288 ± 1.044	0.682

Data are shown as the mean ± SD; * represents a statistically significant difference

### Loss of the PTEN gene and its relationship with the PI3K/AKT/mTOR signaling pathway in PCNSL

**Fig. 4:**
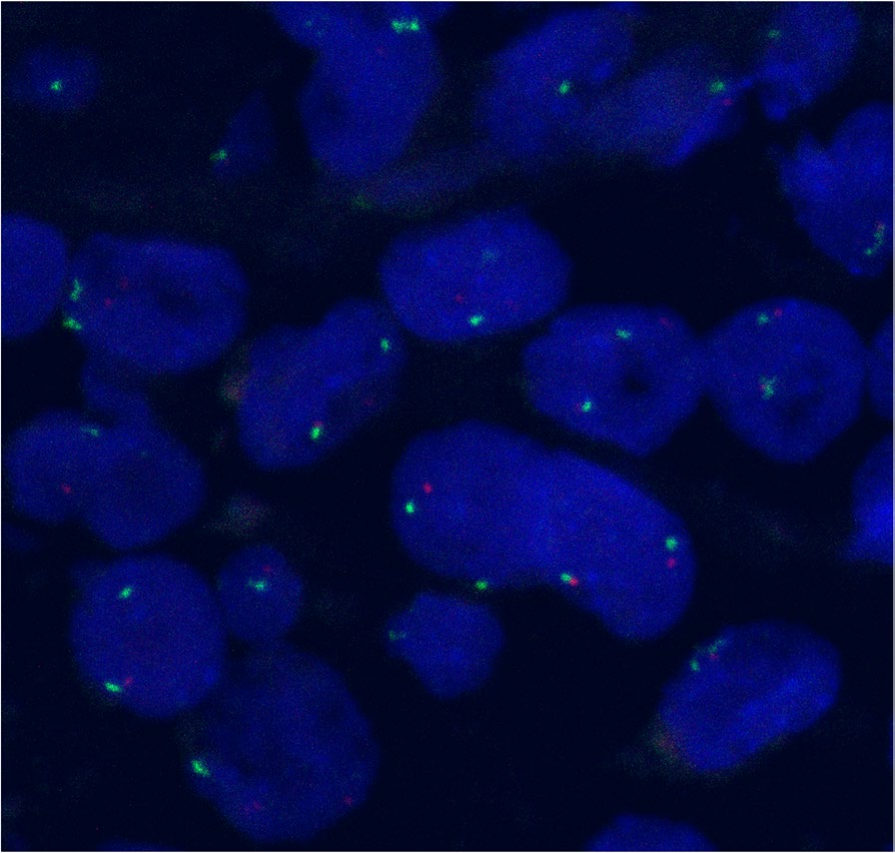
Detection of the loss of *PTEN* by FISH in PCNSL samples (*n* = 37). DAPI nuclear staining is shown as blue fluorescent signal. The red fluorescent signal represents the *PTEN* gene probe, and the green fluorescent signal represents the centromere probe. The ratio of the *PTEN* signal to centromere signal was less than 0.8 indicating the deletion of *PTEN* gene

The status of *PTEN* loss was detected by FISH in 43 FFPE PCNSL samples. Since the staining results of 6 samples were unsatisfactory, a total of 37 samples were available for analysis. The rate of *PTEN* loss was 18.9% (7/37) (Fig. 4). According to the results of *PTEN* detection, 37 samples of PCNSLs were divided into two groups: the PTEN loss group and the normal group. The positive rates of p-AKT, p-mTOR, p-S6 and p-4E-BP1 expression from the immunohistochemical analysis in the two groups are shown in Table 3. The positive rate of p-AKT in the *PTEN* loss group was 100% (7/7), while in the normal group, the positive rate was only 56.7% (17/30). The difference was statistically significant (*P* = 0.038). However, there was no significant difference in the positive rates of other target proteins between the two groups. We used the Spearman rank correlation coefficient to analyze the relationship between *PTEN* loss and p-AKT expression. The results showed that the correlation coefficient was 0.356 and that the *P* value was 0.031, suggesting that *PTEN* loss was correlated with p-AKT expression.

**Table 3:** The expression rates of p-AKT, p-mTOR, p-S6 and p-4E-BP1 in the *PTEN* loss group and normal group

Protein	*PTEN* loss	*PTEN* normal	*P* value
	(*n* = 7)	(*n* = 30)	
p-AKT	100% (7/7)	56.7% (17/30)	0.038*
p-mTOR	100% (7/7)	70.0% (21/30)	0.160
p-S6	100% (7/7)	76.7% (23/30)	0.306
p-4E-BP1	57.1% (4/7)	73.3% (22/30)	0.403

Note: * represents a statistically significant difference

### Clinical characteristics and prognosis of PCNSL patients and their correlations with the PI3K/AKT/mTOR signaling pathway

We collected the clinical characteristics of 43 patients with PCNSL. The median age was 59 years old (range from 16 to 78), and the proportions of males and females were similar (53.5% vs. 46.5%). All the cases were pathologically confirmed as DLBCL, and approximately 83.7% of them were of the nongerminal center B-cell like (non-GCB) subtype. The ECOG score was ≥2 in the vast majority (93.0%) of patients. However, elevated LDH was found in only 7 patients at initial diagnosis. A total of 62.8% of the patients had single lesions, and 74.4% had deep brain involvement, which referred to lesions involving the cerebellum, brainstem, corpus callosum or basal ganglia. After at least 3 cycles of high-dose methotrexate-based induction chemotherapy, the objective response rate (ORR) was 79.1%, and the complete remission (CR) rate was 37.2%. By the last follow-up (median time 18 months), approximately half (48.8%) of the patients had relapsed, and 6 patients had died.

According to the above clinical characteristics, we divided the PCNSL patients into different groups. The protein expression levels of p-AKT, p-mTOR, p-S6, p-4E-BP1 and the loss of *PTEN* in each group are shown in Supplementary Table S2. Since FISH results were only available for 37 patients, the analysis of *PTEN* loss was performed using clinical data from these 37 patients. The results indicated that the rate of relapse in the p-mTOR-positive group was 64.5% (20/31), while that in the p-mTOR-negative group was only 8.3% (1/12). Similarly, the rate of relapse in the p-S6-positive group was significantly higher than that in the p-S6-negative group (58.8% vs 11.1%). The *P* values were 0.001 and 0.021, respectively. We also found that the rate of *PTEN* loss was 66.7% (4/6) in patients with the GCB subtype and only 9.7% (3/31) in patients with the non-GCB subtype, and the difference was significant (*P* = 0.007). There was no significant correlation between the other targets and the clinical characteristics.

**Fig. 5:**
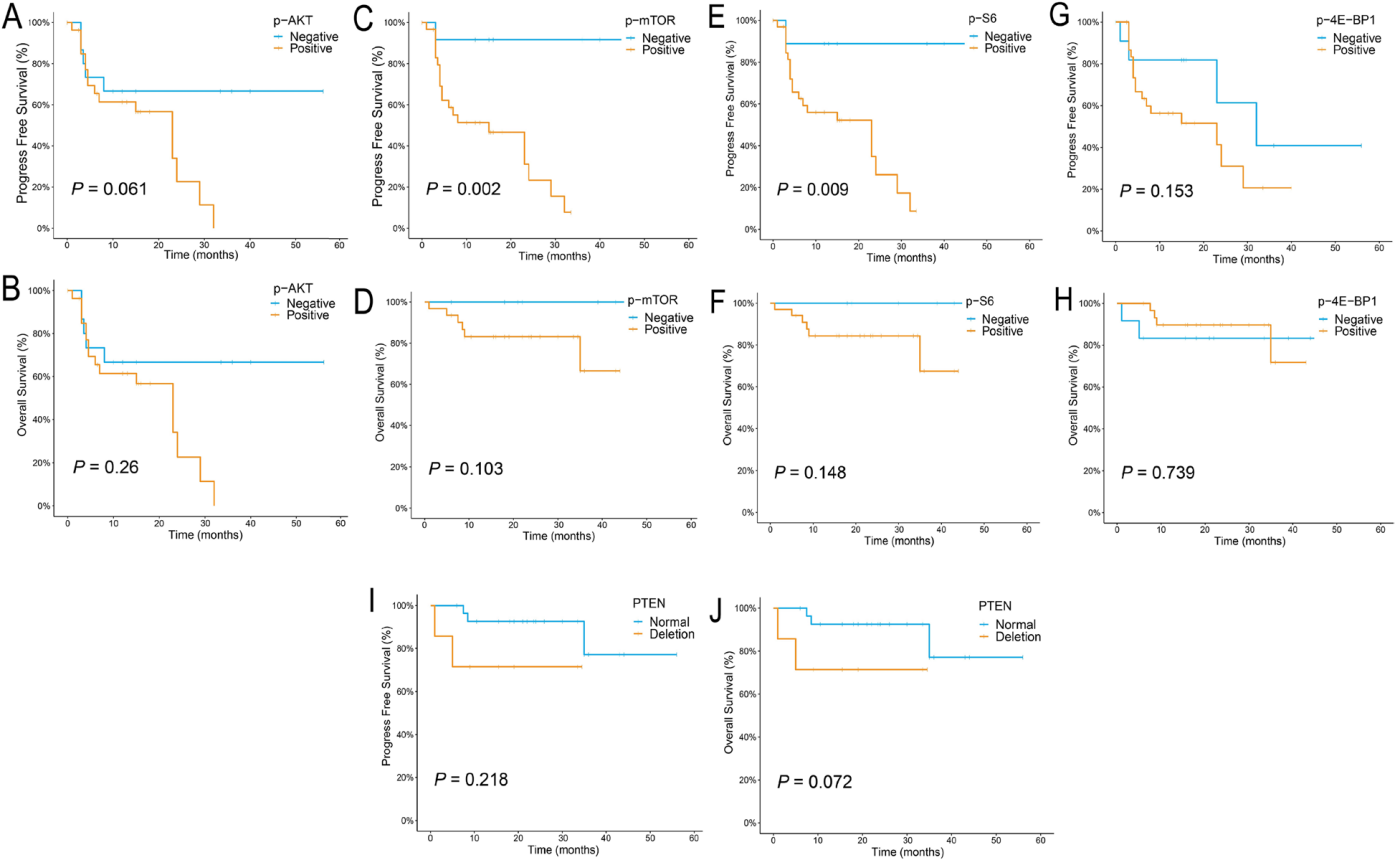
Kaplan-Meier survival curves for OS and PFS according to the expression of p-AKT, p-mTOR, p-S6, and p-4E-BP1 (*n* = 43) and the status of *PTEN* (*n* = 37) in PCNSL cohort

We evaluated the prognosis of PCNSL in terms of OS and PFS. By the end of follow-up, only 6 (14.0%) of the 43 patients had died, so the median OS was not reached. The median PFS calculated by the Kaplan-Meier method was 23 months (95% CI 13.9–32.1 months). The estimated 2-year PFS rate was 40.2%, and the 3-year OS rate was 76.8%. The Kaplan-Meier survival curves for OS and PFS were drawn according to the protein expression levels of p-AKT, p-mTOR, p-S6, and p-4E-BP1 and the loss of *PTEN*, which are shown in Fig. 5. The PFS of the patients with positive p-mTOR or p-S6 expression was significantly shorter than that of the patients with negative expression of the respective proteins, and the estimated 2-year PFS rates were 23.3% vs 97.1 and 26.1% vs 88.9%, respectively, with *P* values of 0.002 and 0.009. (Fig. 5C, Fig. 5E) In addition, Kaplan-Meier analysis also indicated that the OS of the *PTEN* loss group was lower than that of the *PTEN* normal group, with a *P* value of 0.072, close to the statistical significance threshold.

Cox multivariate regression analysis was used to explore the independent risk factors affecting OS and PFS in patients with PCNSL. The including risk factors were as follows: age, sex, ECOG score, LDH level, number of lesions, whether deep brain involvement, pathological subtypes, relapse situation, the protein expression of p-AKT, p-mTOR, p-S6, and p-4E-BP1 and the loss of *PTEN*. The results showed that p-mTOR expression was an independent prognostic factor for a shorter PFS with a hazard ratio (HR) of 7.849 (95% CI: 1.037–59.408) and a *P* value of 0.046.

## Discussion

PCNSL is included in both the WHO Classification of Tumors of Haematopoietic and Lymphoid Tissues and the WHO Classification of Tumors of the Nervous System, indicating the unique features of PCNSL and the extraordinary association of malignant hematopoietic cells with the CNS. However, the CNS lacks conventional lymphatics within the CNS parenchyma. Obtaining intracranial lymphocytes for comparison is not available. Since PCNSL is also a lymphocyte malignant hyperplastic disease, we selected the reactive hyperplastic lymph nodes as the control group to evaluate whether it has specific biological characteristics.

In this study, AKT, mTOR, S6 and 4E-BP1 were selected as targets to explore the role of the PI3K/AKT/mTOR signaling pathway in PCNSL. AKT is one of the major carcinogenic effectors in this signaling pathway, which can be indirectly activated by upstream PI3K and activate downstream mTOR. mTOR comprises mTOR complex 1 (mTORC1) and mTORC2. Activated mTORC1 can continue to activate its downstream S6 and 4E-BP1, while activated mTORC2 can directly phosphorylate its upstream target AKT so that AKT activity is fully activated [32, 33]. After phosphorylation by mTORC1, 4E-BP1 and S6 are able to promote mRNA transcription and protein translation [34].

Our results suggested that the protein expression rates of p-AKT, p-mTOR, p-S6 and p-4E-BP1 in PCNSL samples were significantly higher than those in reactive hyperplastic lymph nodes. The correlation analysis indirectly confirmed the association of their expression levels. In addition, the relative mRNA expression of *MTOR* gene was also abnormally increased. Therefore, we speculated that the PI3K/AKT/mTOR signaling pathway is abnormally activated in PCNSL.

However, Marosvari et al. [35] proposed a different point of view. The positive immunohistochemical staining rate of p-S6 was 58.1% in PCNSL specimens, while that of p-mTOR was only 25.8%, suggesting that the expression of p-S6 might not be induced by mTOR activation. However, another study [36] showed that Rheb, p-4E-BP1 and p-S6 were overexpressed in more than half of PCNSL cases. The results indirectly suggested aberrant activation of mTORC1. In addition, copy number variations (CNVs) in the related genes were detected in PCNSL samples [13], and the frequency of CNVs in PCNSL samples was significantly higher than that in systemic DLBCL, representing greater genomic instability [11]. These results provide evidence for the activation of this pathway in PCNSL.

To further explore the pathological mechanism of the aberrant activation of the PI3K/AKT/mTOR pathway, we detected the loss of the *PTEN* gene in PCNSL samples. *PTEN* is an important negative regulator in this pathway, and its loss can result in the occurrence and development of various malignant tumors [37, 38]. In our study, *PTEN* loss occurred in 18.9% of PCNSL cases and was associated with the expression of p-AKT. Currently, there is no relevant study on the detection of *PTEN* loss by FISH in PCNSL. In systemic DLBCL, some studies found that *PTEN* loss often occurred in the GCB subtype [39]. Furthermore, PI3K inhibitors selectively induced the death of *PTEN*-loss GCB subtype DLBCL cell lines. Although the majority of PCNSLs are non-GCB subtypes, mutations and CNVs in *PTEN* can also be found in the second-generation sequencing analysis [13, 40, 41]. Todorovic et al. [40] found that the mutation rate of *PTEN* was up to 37% and that *PTEN* mutation was related to shorter OS in PCNSL patients. Unfortunately, the effect of *PTEN* loss on the treatment response and prognosis of PCNSL was not further investigated.

We also analyzed the relationship between abnormal activation of the PI3K/AKT/mTOR signaling pathway and the clinical features and prognosis of PCNSL. The results suggested that the positive expression of p-mTOR and p-S6 was significantly correlated with disease recurrence in patients with PCNSL. A study on systemic DLBCL indicated that abnormal activation of this pathway might be correlated with drug resistance to rituximab [42]. Takashima et al. [43] revealed that the PI3K/AKT/mTOR signaling pathway might induce resistance to methotrexate. In addition, Grommes et al. [44] found that PI3K inhibitors combined with ibrutinib could overcome the resistance of PCNSL cells to ibrutinib. Therefore, the mechanism by which abnormal activation of this pathway leads to disease relapse in PCNSL might be related to the acquisition of drug resistance in tumor cells. However, due to the lack of PCNSL cell lines, our experiments on the effects of relevant inhibitors on the activity of PCNSL cells were limited.

In addition, mTOR also participates in regulating autophagy [45, 46]. Autophagy can provide nutrients to tumor cells to promote tumor survival under the condition of limited nutrition. Thus, cancer cells may be sensitized to metabolic stress conditions due to inhibition of autophagy, leading to cell death. Defects in autophagy may enhance genomic instability and contribute to cancer development. Many studies have suggested the role and significance of autophagy in the development of systemic lymphoma [47, 48]. However, only 1 research has provided evidence for the possible existence of autophagy markers in PCNSLs, but has not mentioned the correlation with PI3K/AKT/mTOR signaling pathway [49]. Further investigation is needed to explore this promising issue.

Moreover, we found that the loss of *PTEN* was more common in the GCB subtype of PCNSL and that patients with *PTEN* loss showed a worse OS than those with normal *PTEN*. These findings were consistent with the conclusion of Todorovic et al. [40]. Although the difference in OS between the *PTEN* loss group and the normal group was not statistically significant (*P* = 0.072) in our study, further expansion of the sample size or extension of the follow-up time might increase the significance, and the results would be more convincing.

In the multivariate Cox regression analysis, we found that p-mTOR expression was an independent risk factor in terms of PFS in patients with PCNSL. This result also confirmed the previous assumption that high expression of p-mTOR may be the crucial factor affecting the prognosis of PCNSL. Therefore, for newly diagnosed patients with PCNSL, the detection of p-mTOR is extremely important. On the one hand, we can estimate the risk of relapse to guide the administration of stronger combined immunochemotherapy approaches as needed and strengthen the monitoring of the disease. On the other hand, it also provides a basis for individualized treatment strategies.

We previously reviewed the application prospects of inhibitors targeting the PI3K/AKT/mTOR signaling pathway in PCNSL and found that the efficacy of inhibitors as single agents in the treatment of patients with PCNSL might be limited, and it might be difficult to achieve long-term control of the disease with this approach [50]. Thus, rational combination strategies should be considered, especially for patients with relapsed and refractory PCNSL and newly diagnosed patients who cannot tolerate intensive chemotherapy.

## Conclusions

Our study suggests that the PI3K/AKT/mTOR signaling pathway is aberrantly activated in patients with PCNSL, which may lead to disease relapse and decrease PFS. The loss of *PTEN* may be one of the molecular mechanisms that leads to the aberrant activation of this signaling pathway and may be associated with poor OS. Moreover, our study also provides a theoretical basis for the rational clinical application of inhibitors targeting the PI3K/AKT/mTOR signaling pathway. However, how to select targeted drugs and how to combine them with chemotherapy regimens still need to be further explored in both preclinical and clinical studies.

## Supplementary Information

**Additional file 1**: **Supplementary Table S1**. Primer sequences for qPCR.

**Additional file 2**: **Supplementary Table S2.** Clinical characteristics and prognosis of PCNSL patients and their correlations with the protein expression of p-AKT, p-mTOR, p-S6, p-4E-BP1 and the loss of PTEN.

**Additional file 3**: **Supplementary Figure 1**.

## Data Availability

The datasets analyzed during the current study are available from the corresponding author on reasonable request.

## Abbreviations

BCA: Bicinchoninic acid
BCR: B cell receptor
BSA: Bovine serum albumin
CNS: Central nervous system
CNV: Copy number variation
CR: Complete remission
DAPI: Diamidino-2-phenylindole
DLBCL: Diffuse large B-cell lymphomas
EBV: Epstein-Barr virus
ECOG: Eastern Cooperative Oncology Group
FFPE: Formalin-fixed, paraffin-embedded
FISH: Fluorescence in situ hybridization
GCB: Germinal center B-cell
HR: Hazard ratio
IHC: Immunohistochemistry
LDH: Lactate dehydrogenase
mTORC1: mTOR complex 1
mTORC2: mTOR complex 2
MyD88: Myeloid differentiation factor 88
ORR: Objective response rate
OS: Overall survival
PCNSL: Primary central nervous system lymphoma
PFS: Progression-free survival
PVDF: Polyvinylidene fluoride
SDS: Sodium dodecyl sulfate
SSC: Saline-sodium citrate
