# Supplementary material for: The PI3K/AKT/mTOR signaling pathway is aberrantly activated in primary central nervous system lymphoma and correlated with a poor prognosis
Source: BMC Cancer. 2022 Feb 20;22:190. doi: 10.1186/s12885-022-09275-z (PMC8859899; doi:10.1186/s12885-022-09275-z)
Supplement: Supplementary file 1 — Additional file 1: Supplementary Table S1. Primer sequences for qPCR. [file 12885_2022_9275_MOESM1_ESM.docx]

| **Gene** | **Forward primer** **(5’-3’)** | **Reverse primer (3’-5’)** |
| --- | --- | --- |
| *AKT1* | CTGCACAAACGAGGGGAGTA | GCGCCACAGAGAAGTTGTTG |
| *MTOR* | ATCTTGGCCATAGCTAGCCTC | ACAACTGGGTCATTGGAGGG |
| *RPS6* | TGAAGCAGGGTGTCTTGACC | GCATCCACAATGCAACCACG |
| *EIF4EBP1* | CAAGGGATCTGCCCACCATT | AACTGTGACTCTTCACCGCC |

**Supplementary Table S1.** **Primer sequences for qPCR**
