# Supplementary material for: The PI3K/AKT/mTOR signaling pathway is aberrantly activated in primary central nervous system lymphoma and correlated with a poor prognosis
Source: BMC Cancer. 2022 Feb 20;22:190. doi: 10.1186/s12885-022-09275-z (PMC8859899; doi:10.1186/s12885-022-09275-z)
Supplement: Supplementary file 2 — Additional file 2: Supplementary Table S2. Clinical characteristics and prognosis of PCNSL patients and their correlations with the protein expression of p-AKT, p-mTOR, p-S6, p-4E-BP1 and the loss of PTEN. [file 12885_2022_9275_MOESM2_ESM.docx]

| Characteristics | N=43 | | | | | | | | | | | | N=37 | | |
| --- | --- | --- | --- | --- | --- | --- | --- | --- | --- | --- | --- | --- | --- | --- | --- |
|  | p-AKT | | | p-mTOR | | | p-S6 | | | p-4E-BP1 | | | *PTEN* gene | | |
|  | - | + | P | - | + | P | - | + | P | - | + | P | Loss | Normal | P |
| Total | 15 | 28 |  | 12 | 31 |  | 9 | 34 |  | 12 | 31 |  | 7 | 30 |  |
| Age |  |  |  |  |  |  |  |  |  |  |  |  |  |  |  |
| ＜60 | 8 | 14 | 0.835 | 8 | 14 | 0.206 | 6 | 16 | 0.457 | 8 | 14 | 0.206 | 5 | 15 | 0.416 |
| ≥60 | 7 | 14 |  | 4 | 17 |  | 3 | 18 |  | 4 | 17 |  | 2 | 15 |  |
| Sex |  |  |  |  |  |  |  |  |  |  |  |  |  |  |  |
| Female | 8 | 12 | 0.512 | 4 | 16 | 0.281 | 5 | 15 | 0.711 | 6 | 14 | 0.775 | 4 | 14 | 0.693 |
| Male | 7 | 16 |  | 8 | 15 |  | 4 | 19 |  | 6 | 17 |  | 3 | 16 |  |
| ECOG |  |  |  |  |  |  |  |  |  |  |  |  |  |  |  |
| ＜2 | 1 | 2 | 1.000 | 1 | 2 | 1.000 | 0 | 3 | 1.000 | 0 | 3 | 0.548 | 0 | 3 | 1.000 |
| ≥2 | 14 | 26 |  | 11 | 29 |  | 9 | 31 |  | 12 | 28 |  | 7 | 27 |  |
| LDH |  |  |  |  |  |  |  |  |  |  |  |  |  |  |  |
| Elevated | 1 | 6 | 0.391 | 2 | 5 | 1.000 | 1 | 6 | 1.000 | 1 | 6 | 0.652 | 1 | 4 | 1.000 |
| Normal | 14 | 22 |  | 10 | 26 |  | 8 | 28 |  | 11 | 25 |  | 6 | 26 |  |
| Lesion |  |  |  |  |  |  |  |  |  |  |  |  |  |  |  |
| Single | 8 | 19 | 0.348 | 8 | 19 | 0.744 | 5 | 22 | 0.706 | 9 | 18 | 0.484 | 6 | 20 | 0.694 |
| Multiple | 7 | 9 |  | 4 | 12 |  | 4 | 12 |  | 3 | 13 |  | 1 | 10 |  |
| Deep involvement |  |  |  |  |  |  |  |  |  |  |  |  |  |  |  |
| Presence | 13 | 19 | 0.178 | 9 | 23 | 1.000 | 8 | 24 | 0.407 | 7 | 25 | 0.241 | 4 | 23 | 0.360 |
| Absence | 2 | 9 |  | 3 | 8 |  | 1 | 10 |  | 5 | 6 |  | 3 | 7 |  |
| Subtype |  |  |  |  |  |  |  |  |  |  |  |  |  |  |  |
| GCB | 2 | 5 | 1.000 | 3 | 4 | 0.378 | 1 | 6 | 1.000 | 2 | 5 | 1.000 | 4 | 2 | 0.007* |
| non-GCB | 13 | 23 |  | 9 | 27 |  | 8 | 28 |  | 10 | 26 |  | 3 | 28 |  |
| Relapse |  |  |  |  |  |  |  |  |  |  |  |  |  |  |  |
| Presence | 5 | 16 | 0.137 | 1 | 20 | 0.001* | 1 | 20 | 0.021* | 4 | 17 | 0.206 | 4 | 14 | 0.693 |
| Absence | 10 | 12 |  | 11 | 11 |  | 8 | 14 |  | 8 | 14 |  | 3 | 16 |  |
| Outcome |  |  |  |  |  |  |  |  |  |  |  |  |  |  |  |
| Alive | 14 | 23 | 0.403 | 12 | 25 | 0.163 | 9 | 28 | 0.315 | 10 | 27 | 1.000 | 5 | 27 | 0.233 |
| Dead | 1 | 5 |  | 0 | 6 |  | 0 | 6 |  | 2 | 4 |  | 2 | 3 |  |

**Supplementary Table S2.** Clinical characteristics and prognosis of PCNSL patients and their correlations with the protein expression of p-AKT, p-mTOR, p-S6, p-4E-BP1 and the loss of PTEN.
