# Supplementary material for: The PI3K/AKT/mTOR signaling pathway is aberrantly activated in primary central nervous system lymphoma and correlated with a poor prognosis
Source: BMC Cancer. 2022 Feb 20;22:190. doi: 10.1186/s12885-022-09275-z (PMC8859899; doi:10.1186/s12885-022-09275-z)

**Supplementary Figure 1**

Original images for Figure 2

Control

Control

PCNSL

PCNSL

1

2

3

4

7

6

5

marker

marker

1

2

3

4

6

5

marker

marker

Control

Control

PCNSL

PCNSL

1

2

3

4

7

6

5

marker

marker

1

2

3

4

6

5

marker

marker


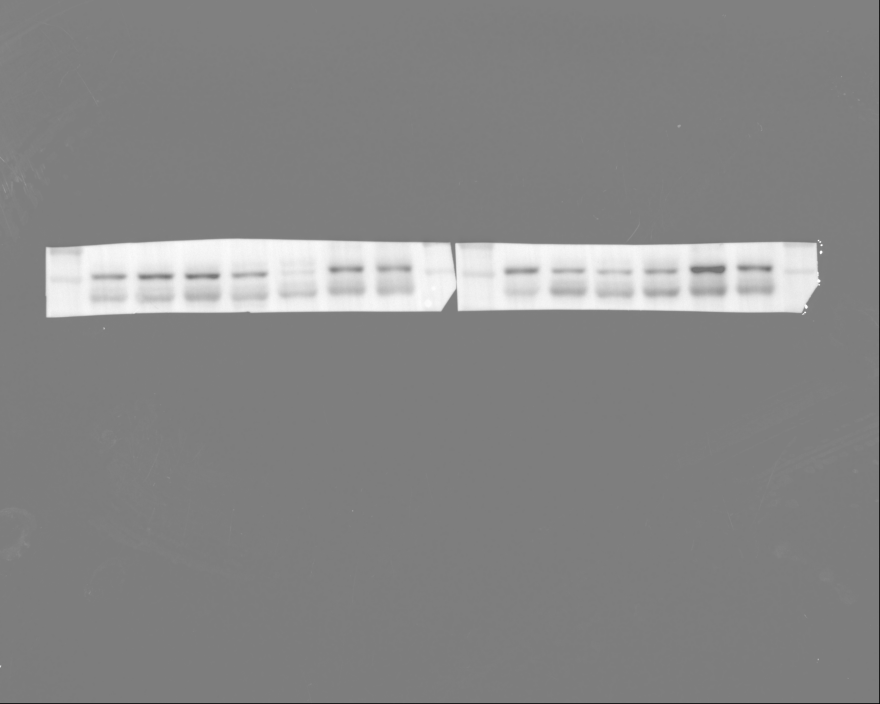


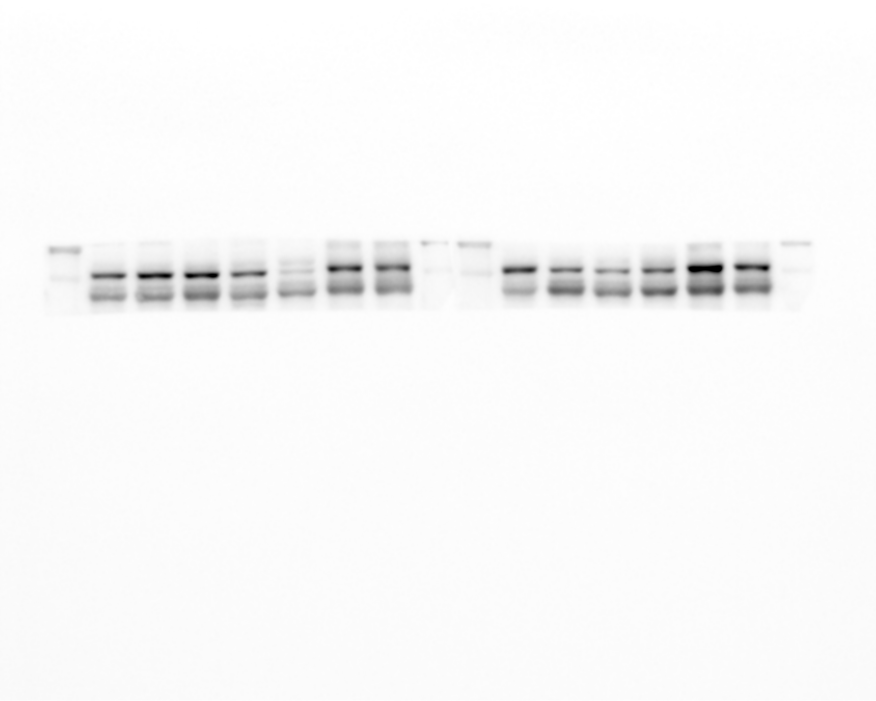


—75—

—63—

—48—

kDa

p-AKT→

(60kDa)

Control

Control

PCNSL

PCNSL

1

2

3

4

7

6

5

marker

marker

1

2

3

4

6

5

marker

marker

Control

Control

PCNSL

PCNSL

1

2

3

4

7

6

5

marker

marker

1

2

3

4

6

5

marker

marker


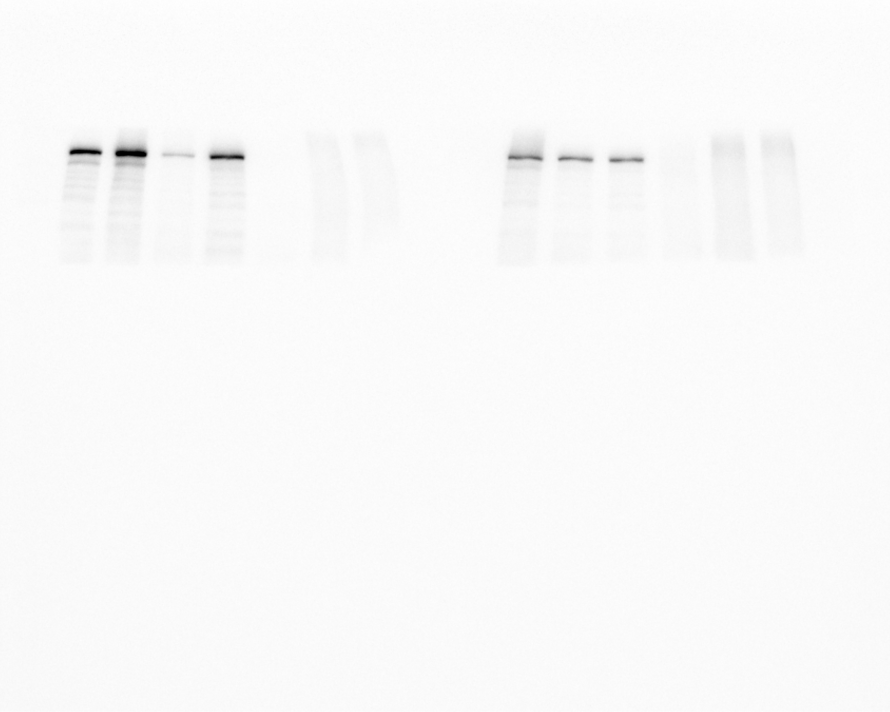

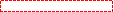

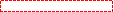


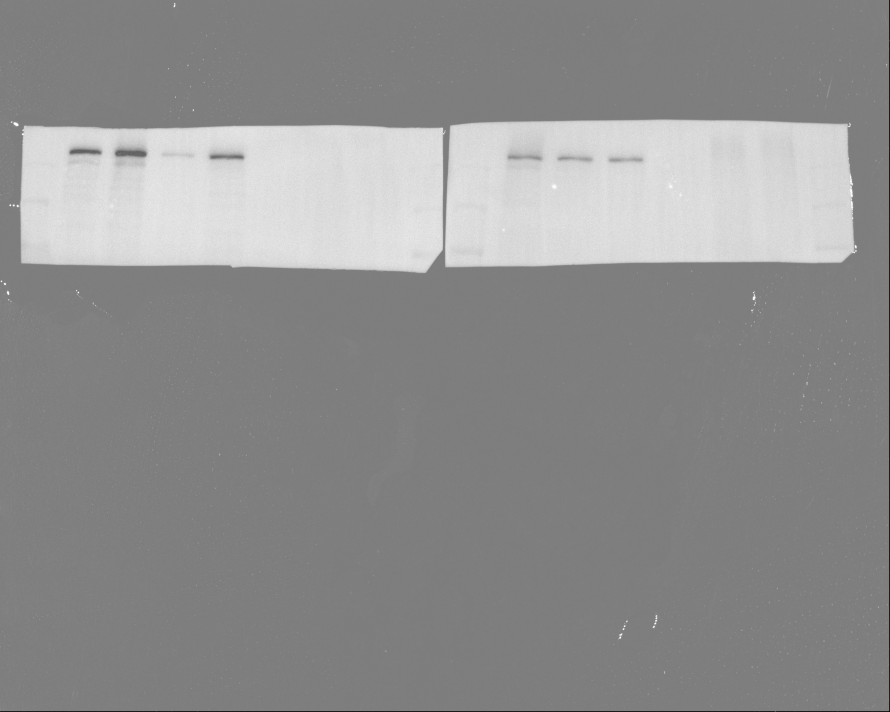


—245—

—180—

—135—

kDa

p-mTOR→

(289kDa)

Control

Control

PCNSL

PCNSL

1

2

3

4

7

6

5

marker

marker

1

2

3

4

6

5

marker

marker

Control

Control

PCNSL

PCNSL

1

2

3

4

7

6

5

marker

marker

1

2

3

4

6

5

marker

marker


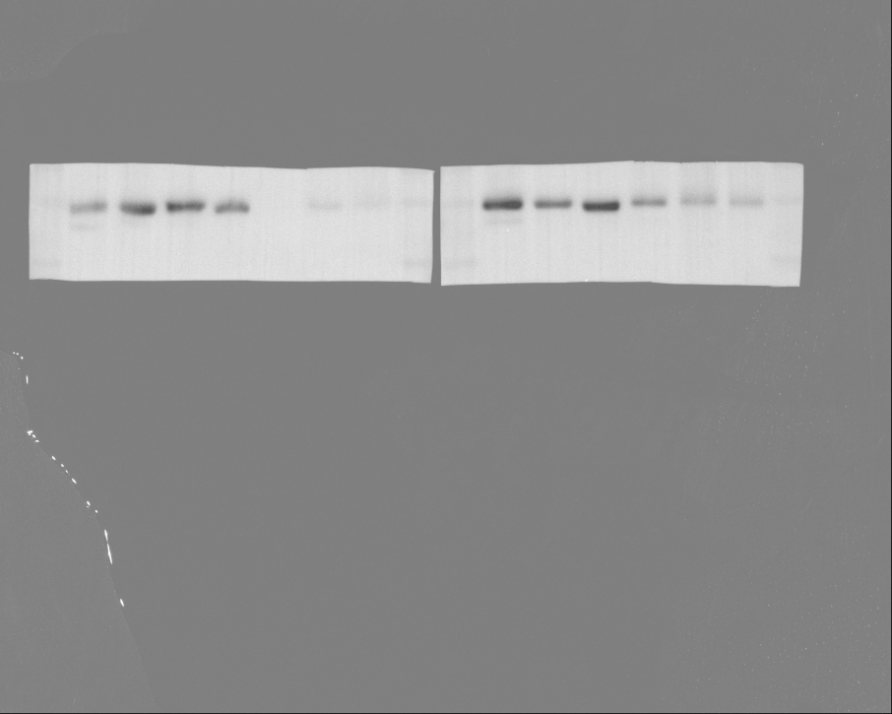

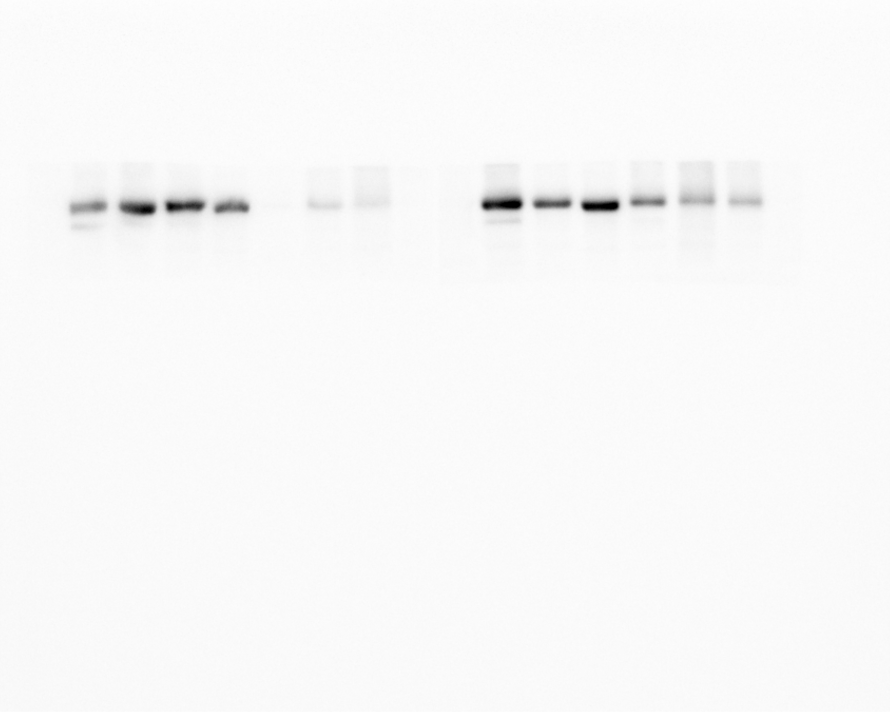


kDa

—48—

—35—

—25—


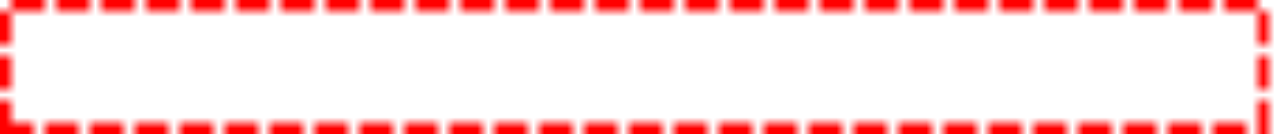

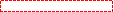


p-S6→

(32kDa)

Control

Control

PCNSL

PCNSL

1

2

3

4

7

6

5

marker

marker

1

2

3

4

6

5

marker

marker

Control

Control

PCNSL

PCNSL

1

2

3

4

7

6

5

marker

marker

1

2

3

4

6

5

marker

marker


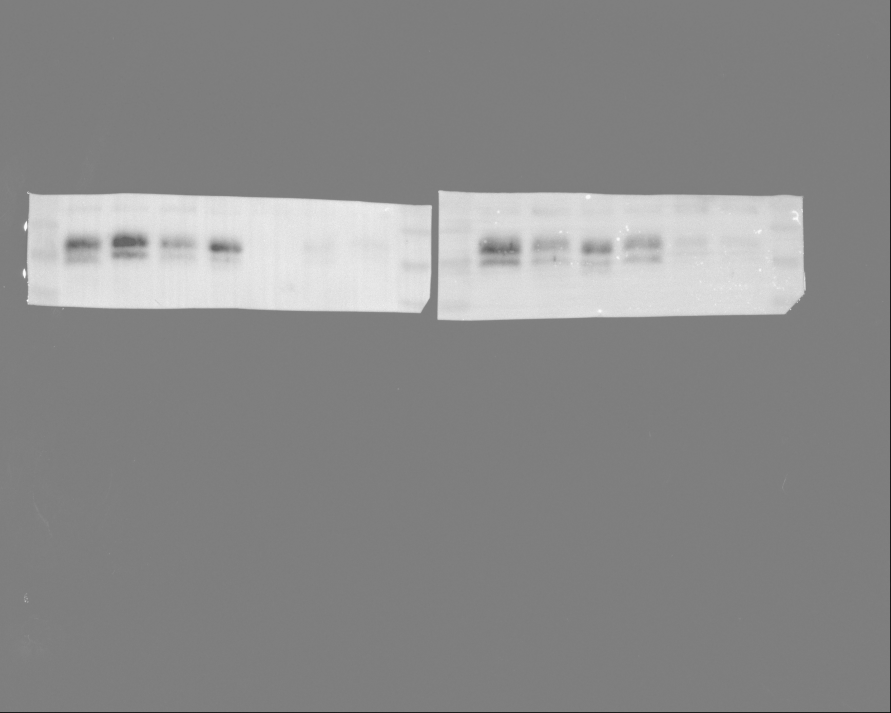

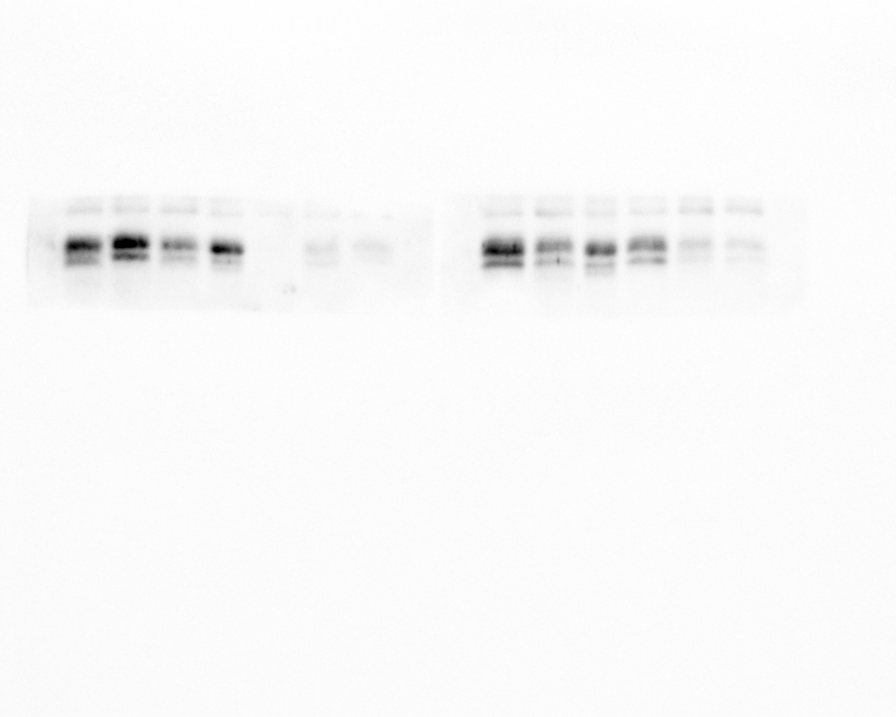


—20—

kDa

—25—

—11—

—17—

p-4E-BP1→

(15-20kDa)


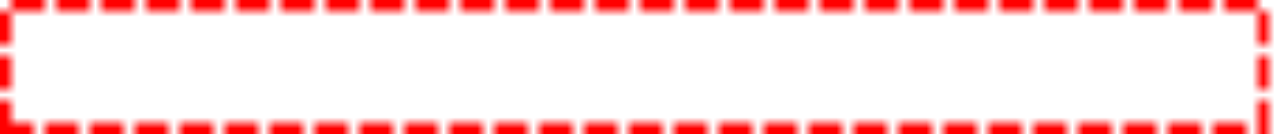

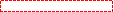


Control

PCNSL

1

2

3

4

7

6

5

marker

marker

Control

PCNSL

1

2

3

4

7

6

5

marker

marker


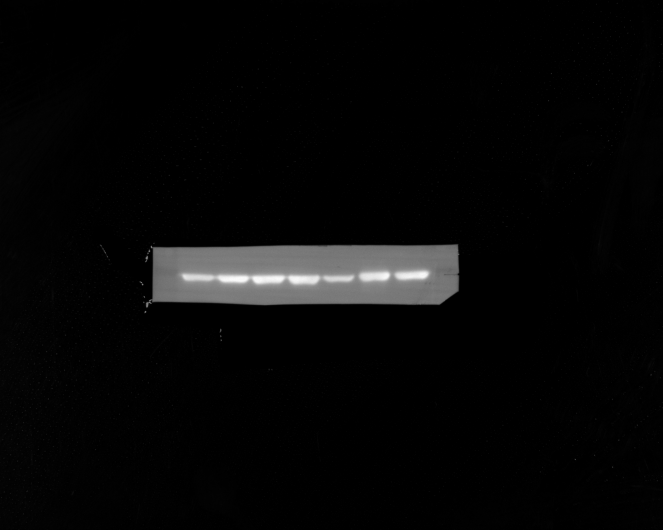

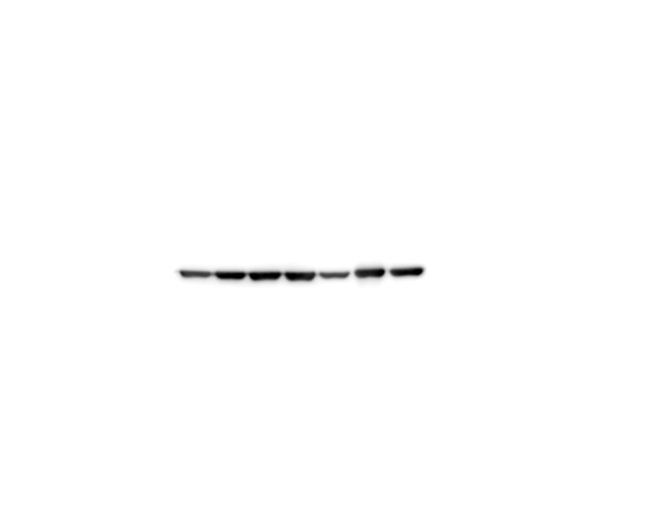


—63—

—48—

kDa

—35—

β-actin→

(43kDa)


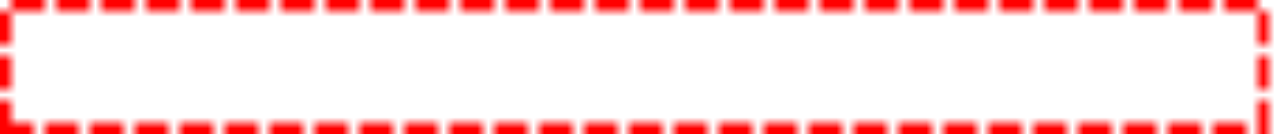


Control

PCNSL

1

2

3

4

6

5

marker

marker

Control

PCNSL

1

2

3

4

6

5

marker

marker


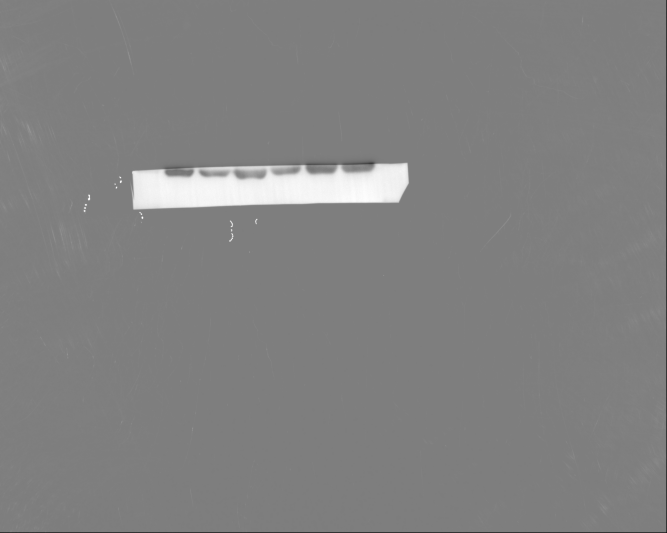

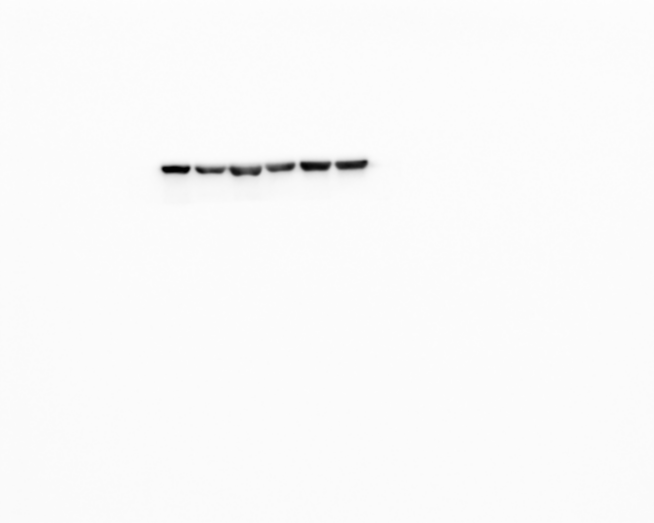


kDa

—48—

—35—

β-actin→

(43kDa)


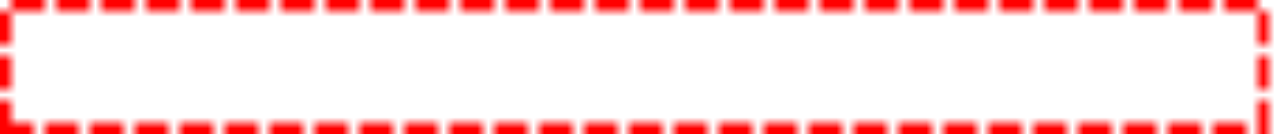

Supplement: Supplementary file 3 — Additional file 3: Supplementary Figure 1. [file 12885_2022_9275_MOESM3_ESM.docx]
